# A Case of Sphenoid Sinusitis With Epidural Abscess due to Fungal Infection and Mixed Infection With *Eikenella corrodens* and *Aggregatibacter segnis*

**DOI:** 10.1155/crot/7328742

**Published:** 2025-08-17

**Authors:** Momotaro Harano, Shiori Tanaka, Kohei Inomata, Shoji Naito, Hidenori Yokoi

**Affiliations:** ^1^Department of Otolaryngology, Kyorin University Suginami Hospital, Tokyo, Japan; ^2^Department of Otolaryngology, Head and Neck Surgery, Kyorin University School of Medicine, Tokyo, Japan

**Keywords:** *Aggregatibacter segnis*, *Eikenella corrodens*, epidural abscess, rhinogenic intracranial complication, sphenoid sinusitis

## Abstract

We report a case of sphenoiditis with an epidural abscess due to a fungal infection and a mixed infection with *Eikenella corrodens* and *Aggregatibacter segnis* and its clinical course, including a literature review. The patient was a 60-year-old woman who visited the emergency department of our hospital with complaints of a sudden right-sided headache that had persisted for 5 days and vomiting for 1 day. She was admitted to the hospital for an examination. Blood test results indicated a strong inflammatory reaction, and a blood culture revealed Gram-negative bacilli. Based on computed tomography and magnetic resonance imaging findings, right sphenoid sinus mycosis and a right-sided epidural hematoma were suspected, and therefore, endoscopic sinus surgery was performed. Thinning and pulsation of the right lateral wall of the sphenoid sinus were noted intraoperatively. The patient also had bacteremia, and the epidural hematoma was considered to be an abscess. The patient's symptoms improved postoperatively. Thus, she was discharged 20 days following the surgery after continuous administration of meropenem for the right-sided epidural abscess. Although cases of epidural abscesses due to infection of the sphenoid sinus are rare, in patients with severe headaches, the presence or absence of intracranial spread at an early stage must be considered to accurately diagnose and treat the pathology.

## 1. Introduction

Rhinosinusitis is a rare intracranial complication that can be serious if the diagnosis and treatment are delayed. In recent years, with the development of antimicrobial agents, the disease has become rare [1, 2]. Herein, we report a case of a patient with an epidural abscess secondary to a mixed infection with the rare species *Eikenella corrodens* and *Aggregatibacter segnis*, which were detected in blood culture, and a fungal infection of the sphenoid sinus. Here, we report the clinical manifestations and treatment course in this case along with a literature review.

## 2. Case Presentation

**Figure 1:**
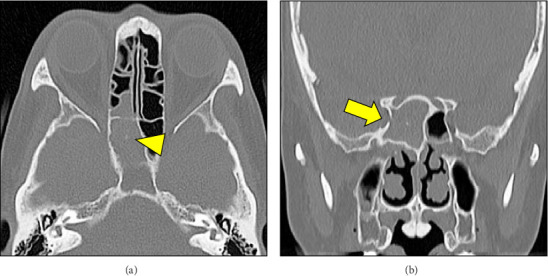
A plain computed tomography scan showing soft-tissue opacifications filling the right sphenoid sinus, calcification within the sinus (yellow arrowhead) (a), and bone thinning outside the sinus (yellow arrow) (b), raising the suspicion of mycosis fungoides of the right sphenoid sinus.

**Figure 2:**
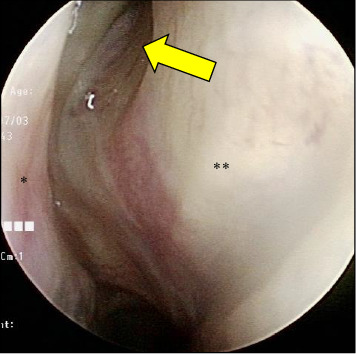
Nasal examination showing purulent nasal discharge from the right sphenoid sinus natural ostium. ^∗^Lower end of middle nasal turbinate, ^∗∗^Septum.

A 60-year-old woman visited the emergency room of our hospital with complaints of a worsening sudden-onset headache that had persisted for 5 days and vomiting the day before. She also experienced chills and pain from the right side of her head to the depth of her right eye. A blood test showed a white blood cell (WBC) count of 6100/μL and an elevated C-reactive protein (CRP) level of 4.93 mg/dL. The patient had a mild fever (temperature, 37.2°C) and a clear consciousness; there was no posterior rigidity or abnormal neurological findings. Although there were no abnormalities in visual acuity, the visual field, or intraocular pressure, the headaches worsened. Plain computed tomography (CT) showed soft-tissue opacifications in the right sphenoid sinus, calcification within the sinus, and bone thinning outside the sinus (Figures 1(a) and 1(b)), raising the suspicion of mycosis fungoides of the right sphenoid sinus. The patient was admitted to the otolaryngology department for further evaluation and treatment. Her medical history included hypothyroidism, postoperative left breast cancer, and dyslipidemia; there was no obvious evidence of immunodeficiency. A nasal examination revealed purulent nasal discharge from the natural ostium of the right sphenoid sinus (Figure 2), and a nasal discharge culture revealed *Klebsiella aerogenes* and coagulase-negative staphylococci.

**Figure 3:**
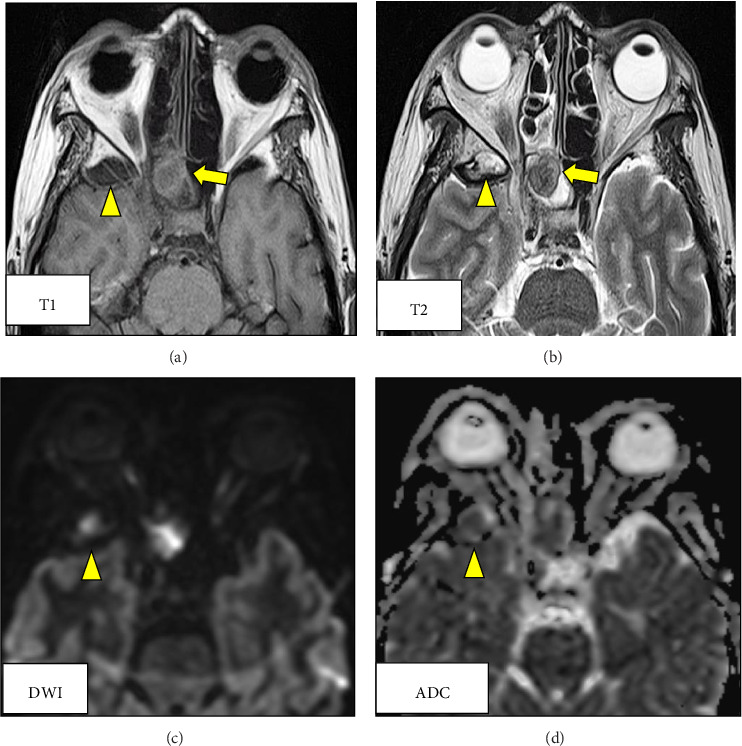
Magnetic resonance imaging scan showing an epidural hematoma in the right middle cranial fossa (yellow arrowhead) and findings indicative of mycosis fungoides the right sphenoid sinus (yellow arrow) (a, b). T1-weighted imaging showing high- and low-intensity areas in the peripheral and internal regions of the right sphenoid sinus, respectively (a). T2-weighted imaging showing low-intensity areas of the right sphenoid sinus on (b) (yellow arrow). T2-weighted imaging (b) and diffusion-weighted imaging (c) showing high intensity, and the apparent diffusion coefficient (ADC) (d) shows low intensity of center (yellow arrowhead).

**Figure 4:**
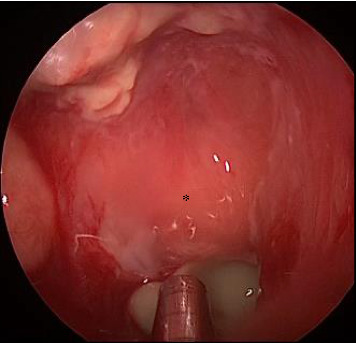
Intraoperative findings: white contents in the sphenoid sinus (yellow head) and a partially thinned area on the lateral aspect of the right sphenoid sinus; furthermore, pulsation was confirmed (yellow arrow). ^∗^Pituitary fossa.

**Figure 5:**
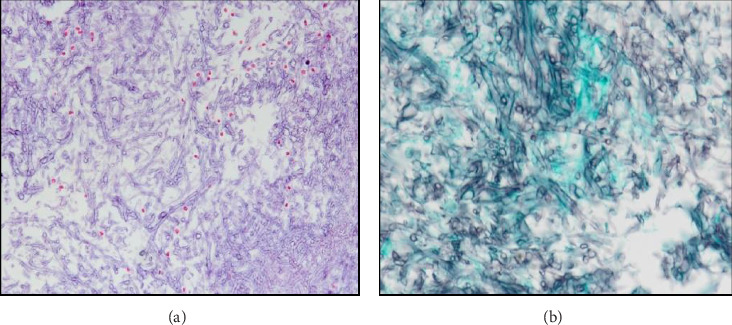
Pathologic examination showing sphenoidal sinus with a segmented wall and branched mycelium suggestive of *Aspergillus*: (a) Periodic acid–Schiff staining. (b) Grocott staining.

**Figure 6:**
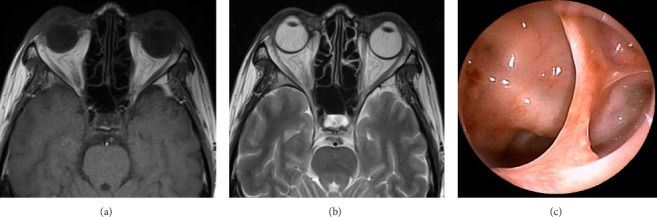
Postoperative magnetic resonance imaging showing an absence of lesions in the sphenoid sinus and the right middle cranial fossa (a, b) and intranasal examination showing no abnormal findings in the sphenoid sinus (c).

The patient's headache continued to worsen, and blood test findings were as follows: WBC count, 8700/μL; a normal β-D-glucan level, and further elevation of CRP (7.94 mg/dL). Tazobactam/piperacillin infusion was started; blood culture showed *Eikenella corrodens*; and *Aggregatibacter segnis*. Magnetic resonance imaging (MRI) revealed an epidural hematoma in the right middle cranial fossa and raised the suspicion of mycosis fungoides of the right sphenoid sinus (Figures 3(a), 3(b), 3(c), 3(d)). Testing of blood samples obtained on the 12th hospitalization day revealed a WBC count of 4000/μL and the CRP level of 0.1 mg/dL, indicating improvement. However, her headache was not alleviated. Therefore, endoscopic sinus surgery was performed under general anesthesia on the 15th day of hospitalization, and the sphenoid sinus was opened wide. Intraoperatively, white contents were noted in the sphenoid sinus, which were removed. A partially thinned area was observed on the lateral aspect of the right sphenoid sinus, and pulsation was confirmed (Figure 4). Pathological examination revealed sphenoidal sinus content with a segmented wall and branched mycelia suggestive of *Aspergillus* (Figures 5(a) and 5(b)). The intraoperative findings of sphenoid sinus wall thinning and blood culture results suggested an epidural abscess in the right middle cranial fossa. The antibiotic was changed to meropenem (MEPM), a drug with a broad spectrum of antibacterial activity in vitro and to which the majority of Gram-negative, Gram-positive, and anaerobic pathogens are highly susceptible. A repeat blood culture performed after 20 days of treatment was negative, and the patient was discharged without headaches. A CT performed 1 month after the surgery showed that the soft-tissue opacifications in the sphenoid sinus had disappeared and the epidural abscess had shrunk. Four months after surgery, the patient was still symptom-free; an MRI showed no lesion in the sphenoid sinus and right middle cranial fossa, and an intranasal examination showed no abnormal findings in the sphenoid sinus (Figures 6(a), 6(b), 6(c)). Four years have passed since, and there has been no recurrence.

## 3. Discussion

Rhinosinusitis is an infectious disease that spreads from the sinuses to the central nervous system through direct invasion by vascular and bone destruction [1]. Although the incidence of nasal intracranial complications has decreased owing to the widespread use of antibacterial agents, attention must be paid because they can rapidly develop into serious conditions. Over the past 20 years, the mortality rate of rhinosinusitis has been reported to be 0%–7%, and the rate of residual disease in survivors is 10%–25%, indicating the need for an appropriate diagnosis and prompt treatment [1].

Although sinusitis is most commonly caused by *Streptococcus pneumoniae*, *Haemophilus influenzae*, *Staphylococcus aureus*, and *Moraxella catarrhalis* [3, 4], the causative organisms of intracranial complications are diverse and include anaerobic bacteria associated with dental infections [5]. Additionally, several cases caused by infection with fungi such as *Aspergillus* and *Mucorales* have been reported [6, 7]. Furthermore, Basidiomycetes, which are filamentous fungi, have been reported to be major pathogens of fungal central nervous system infection associated with sinusitis [7–9]. In the present case, right-sided sphenoid sinus mycosis was observed, and nasal discharge culture revealed a common fungal infection of the sphenoid sinus. Nasal culture showed *Klebsiella aerogenes* and coagulase-negative staphylococci, while blood culture showed *E. corrodens* and *A. segnis*, which are rare species. MRI and subsequent surgical findings indicated that the patient had right-sided sphenoid sinus mycosis and an epidural abscess at the base of the right temporal lobe. To our knowledge, this is the first such case to be reported.

In previous reports of intracranial sinusitis complications, the frontal sinus was the main site of involvement. It has long been thought that the frontal sinus mucosa is separated from the interosseous veins by a very thin bony wall and that the mucosal veins have no valves, allowing the mucosal veins in the sinus to communicate freely with interosseous and dura mater veins and that inflammation in the frontal sinus spreads hematogenously intracranially [10]. However, the findings in the present case suggested a chronic fungal infection. Although no ocular symptoms were observed owing to only a slight thickening of the sphenoid bone, an area of bone thinning was observed in the right lateral inferior part of the bone, suggesting that acute inflammation in the same area had developed subdurally.

Brain abscess symptoms are nonspecific and often include headache, fever, nausea and vomiting, neurological deficits, altered mental status, and seizures. Fever, headache, and focal symptoms are the classic triad; however, only 20% of patients have all the symptoms, and inflammatory factors, such as leukocyte count and the CRP level, may be normal. Therefore, general blood tests are not useful for diagnosis [11].

*E. corrodens*, which was noted in the blood culture in the present case, is a Gram-negative, short rod that is endemic to the nasal, oral, and gastrointestinal tracts and has recently been reported as the causative organism of abscess lesions in the head, neck, and chest areas [12–14]. In such cases, the infection is often caused by multiple bacteria, and, as in the present case, the other causative organisms are often oral streptococci [12, 13]. Therefore, careful isolation and culture of the isolate are essential for the identification of the causative organism of the lesions.

Animal studies have shown that *E. corrodens* does not form a subcutaneous abscess when inoculated alone but forms a subcutaneous abscess when inoculated with oral streptococci [14] and that the presence of *E. corrodens* accelerates the growth of streptococci in vitro [15]. Despite reports that some *E. corrodens* strains produce beta-lactamase [16], most are susceptible to ampicillin. However, *E. corrodens* is resistant to macrolides, aminoglycosides, and first-generation cephems; hence, care must be taken when selecting antimicrobial agents. Careful isolation and culture of the isolate are essential for the identification of the causative organism of the lesions.

We reported a case of spontaneous sinusitis without any surgery or trauma history. The infection source was the sphenoid sinus. Imaging and intraoperative findings revealed thinning of the sphenoid sinus wall, leading us to suspect a bone defect. In addition, blood cultures revealed bacteria, suggesting that inflammatory spillover from the vasculature may also be involved. Initially, the intracranial lesion was thought to be an epidural hematoma, and the patient was treated with antibiotics. However, given that inflammation often spreads through the vasculature, it may have been desirable to consider an epidural abscess at an early stage before initiating treatment. Nasal intracranial complications are often difficult to treat with antimicrobial agents alone, and it is necessary to consider opening the sinus cavity, which is the infection source, and draining the abscess using neurosurgery. These complications can be treated conservatively if there are no central nervous system issues such as impaired consciousness. However, if severe symptoms develop, early sinus surgery and neurosurgical treatment of the intracranial lesion are necessary.

In the present case, there were no neurological findings other than headache and vomiting at the time of admission, and the neurosurgeon did not find any emergent intracranial lesions. However, since the patient's symptoms did not improve, sinus surgery was performed to reduce inflammation spread, and the patient's symptoms were alleviated. Sinus surgery is considered effective for infection control.

## Data Availability

The data that support the findings of this study are available on request from the corresponding author.
